# The impact of cooling and Moringa supplementation on oxidative stress in serum and milk, including milk cytokines, in heat stressed lactating sows and their litters

**DOI:** 10.1093/tas/txae156

**Published:** 2024-11-13

**Authors:** Wonders O Ogundare, Linda M Beckett, Leriana G Reis, McKeeley C Stansberry, Sydney N Roberts, Uchenna Y Anele, Allan P Schinckel, Theresa M Casey, Radiah C Minor

**Affiliations:** Department of Animal Sciences, West Lafayette, IN 47907, USA; Department of Animal Sciences, West Lafayette, IN 47907, USA; Department of Animal Sciences, West Lafayette, IN 47907, USA; Department of Animal Sciences, West Lafayette, IN 47907, USA; Department of Animal Sciences, Greensboro, NC, 27411, USA; Department of Animal Sciences, Greensboro, NC, 27411, USA; Department of Animal Sciences, West Lafayette, IN 47907, USA; Department of Animal Sciences, West Lafayette, IN 47907, USA; Department of Animal Sciences, Greensboro, NC, 27411, USA

**Keywords:** cooling pad, heat stress, Moringa, oxidative stress, sow, lactation

## Abstract

Heat stress (HS) poses a significant challenge to the United States swine industry. Sows and their piglets are particularly vulnerable to HS, as the periparturient phase is characterized by heightened metabolism and increased oxidative stress and inflammation. The study examined the effects of using conductive electronic cooling pads (ECP) and dietary supplementation with 4% Moringa (M) leaf powder on controlling oxidative stress and inflammation caused by HS in sows and their piglets. Forty-eight late gestation sows were assigned to four treatment groups: HS-fed corn–soybean meal (HS + CS), ECP-fed corn–soybean meal (ECP + CS), HS + M, and ECP + M. Blood was collected from sows on gestation (G) day 112, and lactation (L) day 14 and L20, and from piglets (2 males and 2 females) in each litter on postnatal (PN) day 1 and PN20. Colostrum was collected within 2h of birth of the first neonate, and mature milk was collected on L14. Piglet fecal samples were collected on PN14 to measure calprotectin concentration as a marker of intestinal inflammation. Biological antioxidant potential (BAP), derivatives of reactive oxygen metabolites (dROMs). and oxidative stress index (OSi) were measured in blood and milk samples using a Free Radical Elective Evaluator. Milk samples pooled by day of lactation and treatment group were analyzed using cytokine array. Levels of inflammatory cytokines in colostrum were affected by Moringa supplementation and cooling, but not mature milk. Notably, the anti-inflammatory cytokines interleukin (IL)-10 and IL-1ra were 2.14 and 1.57 Log_2_ higher in the colostrum of HS + M compared to other groups. The OSi of colostrum was higher (*P* = 0.0002) than mature milk. Level of BAP in sow serum was greater in ECP + CS and HS + M (*P* = 0.0291) compared to other groups. Moringa had an overall effect of increasing dROMs (*P* = 0.0035) and levels of OSi were lowest in ECP + CS (*P* = 0.0296) sow serum. Treatments did not affect piglet serum oxidative index (*P* > 0.05) or calprotectin levels (*P* > 0.05). Findings support further studies to investigate the efficacy of using ECP and Moringa supplementation to mitigate inflammation and oxidative stress imposed by heat stress conditions in lactating sows.

## Teaser

Cooling and Moringa have the potential to reduce heat stress-induced oxidative damage and inflammatory cytokine responses in lactating sows. Implementing mitigation strategies, especially around parturition, may be the most effective approach to mitigate the impact of heat stress on sow colostrum quality and piglet performance.

## Graphical Abstract

**Graphical Abstract:**
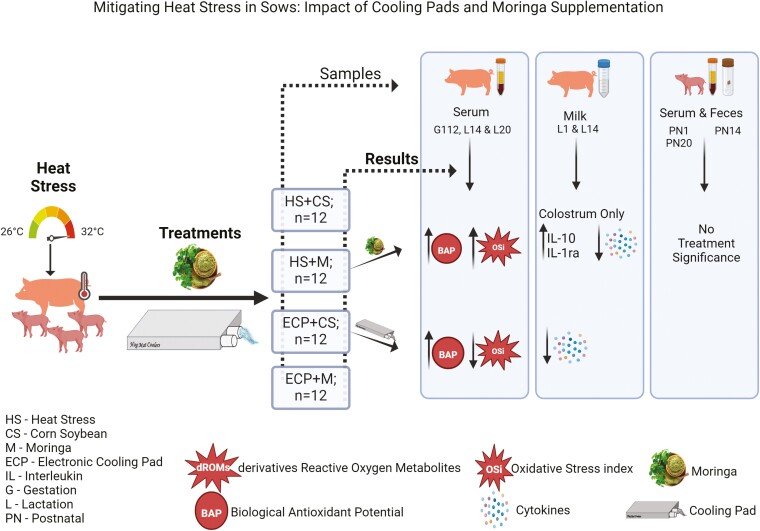

## Introduction

Universally, swine production is a thriving industry with pork ranking as one of the most consumed meats globally. One of the challenges facing the industry stem from negative impacts imposed by heat stress (HS). In the United States alone, economic losses associated with HS are estimated to be approximately $1billion annually (Mayorga et al., 2019). Lactating sows are particularly vulnerable to HS due to the increased metabolic activities associated with the production phase (Lucy and Safranski, 2017).

Inflammation and oxidative stress, marked by an unfavorable ratio of oxidants to antioxidants, are independently associated with lactation phase in sows regardless of presence or absence of HS conditions (Gessner et al., 2015; Li et al., 2020). With HS conditions, oxidative stress is heightened and during the periparturient period, HS exacerbates the naturally occurring oxidative stress and inflammation concomitant with this energy-intensive period. Oxidative stress triggers the release of inflammatory cytokines such as interleukin (IL)-6, IL-1β, and TNF-α while reducing anti-inflammatory cytokines like IL-10 and IL-1ra which contribute to compromise the overall immune status (Pizzino et al., 2017; Lauridsen, 2019; Chen et al., 2021). Oxidative stress increases the damage rate of free radicals against membrane lipids and those that surround the milk fat globules (Belhadj Slimen et al., 2016), and inflammatory responses produce inflammatory cytokines that further propel oxidation reactions. These processes release harmful byproducts that if incorporated into milk of sows may contribute to the detrimental effects of HS on nursing piglets (Devillers et al., 2011).

Conductive cooling of sows using electronic cooling pads (ECP) installed on the floor of farrowing crates, targeting only the sow, has been shown to mitigate the negative impacts of HS during lactation (Cabezón et al., 2017; Parois et al., 2018; Johnson et al., 2022), without increasing risk of hypothermia of neonates. An additional approach to mitigating the negative impact of HS is the use of antioxidant feed supplements. These feed supplements, like Chitosan (Zheng et al., 2021) and *Forsythia suspensa* extract (Long et al., 2021) can mitigate HS-induced oxidative damage in livestock, and improve oxidative balance and inflammatory response of cytokines (Gessner et al., 2017). The *Moringa oleifera* plant, used in this study, is a rich source of antioxidant and anti-inflammatory compounds (Trigo et al., 2020). Ground Moringa leaves given orally to rabbits, extracted into tea for mice, and supplemented with the diet of sows have been shown to alleviate inflammation and oxidative stress (Mcknight et al., 2014; Khalid et al., 2020; Sun et al., 2020). Therefore, in addition to cooling, Moringa leaf powder dietary inclusion may be a potential means of mitigating the negative impacts of HS on oxidative status and inflammation in sows.

We hypothesized that supplementing feed with Moringa leaf powder, and cooling sows exposed to a HS environment in late gestation and throughout lactation would improve maternal inflammation and oxidation status and ultimately positively affect their piglets. Here, we describe the findings of a 2 by 2 factor designed study aimed at assessing the effect of cooling sows with ECP and Moringa supplementation alone or combined on sow milk inflammatory cytokine levels and oxidative status, sow and piglet serum oxidative status, and piglet intestinal health from late gestation to lactation under HS conditions.

## Materials and Methods

### Ethics and Approval

The study was performed at the Swine Unit of Purdue University Animal Research and Education Center (ASREC) in West Lafayette, Indiana, following review and approval of protocol by the Institutional Animal Care and Use Committee (Purdue IACUC Protocol #2110002202).

### Animals, Experimental Design, and Project Overview

This study was part of a larger study previously described (Stansberry et al., 2024). In brief, 48 primiparous and multiparous Large White-Landrace crossed sows, bred to Duroc terminal sires, were enrolled in a 2 × 2 factorial treatment experiment of 4 groups by blocking for parity. Treatments included: HS-fed control corn–soybean meal diet (HS + CS; *n* = 12), HS-fed control diet plus 4% dried Moringa leaf powder (HS + M; *n* = 12), electronic cooling pad + control diet (ECP + CS; *n* = 12), and electronic cooling pad plus Moringa (ECP + M; *n* = 12). The experiment was conducted in two replicates from gestation (G) day 100 ± 2 until lactation (L) day 21 with an equal number of sows (*n* = 24) per replicate. For each treatment, there were *n* = 6 primiparous and *n* = 6 multiparous sows (5 ± 1.5 parity). The first replicate started on August 14, 2022, while the second replicate began on October 12, 2022. Sows were housed in crates throughout the study.

Research diet feeding began G100, which was 9 to 10 d before applying experimental HS exposure. The corn–soybean meal base-control diet was formulated to meet or exceed NRC requirements. Dried *M. oleifera* leaf powder (Jimfina Farm; Brown Summit NC, USA) was added at 4% of the corn–soybean meal diet, and the diet was formulated to have equal ME content (Stansberry et al., 2024). Diets were formulated to meet gestation and lactation requirements (Table 1). Sows were fed gestation diets until approximately G110 and then switched to lactation diets. During gestation, sows were limit-fed by dispersing two meals of 1.36 kg to a total of 2.72 kg/d. After farrowing sows were given ad libitum access to feed. Approximately 48 h after farrowing, litters were standardized to *n* = 13 piglets per sow by cross-fostering within treatments; piglets were maintained within treatment groups of birth sows.

**Table 1.** Gestation and lactation diet composition (as-fed basis)^1^

	Gestation		Lactation	
Item	Control	Moringa	Control	Moringa
*Ingredient, %*				
Corn	79.6	75.2	58.8	54.2
Soybean meal	15.3	14.5	34.0	33.1
Moringa dried leaf powder	0.00	4.00	0.00	4.00
Swine grease	1.00	2.38	3.00	4.70
Limestone	1.31	1.12	1.43	1.23
Monocalcium phosphate	1.06	1.08	1.34	1.37
Phytase	0.10	0.10	0.10	0.10
Provimi sow vitamin + TM premix	0.15	0.15	0.15	0.15
Choline chloride (60%)	0.10	0.10	0.10	0.10
Rovimix-CarniChrom	0.01	0.01	0.01	0.01
Salt	0.50	0.50	0.50	0.50
Availa sow	0.08	0.08	0.08	0.08
Clarify	0.33	0.33	0.10	0.10
Defusion plus/myco prevent	0.25	0.25	0.25	0.25
Titanium premix	0.20	0.20	0.20	0.20
Total	100	100	100	100
*Calculated analysis*				
Metabolizable energy, Kcal/kg	3,293	3,293	3,359	3,360
Crude protein, %	13.9	14.1	21.1	21.2
Total lysine, %	0.65	0.67	1.15	1.17
SID lysine, %	0.55	0.55	1.00	1.00
Calcium, %	0.75	0.75	0.9	0.90
Total phosphorus, %	0.54	0.54	0.68	0.68
ATTD phosphorus, %	0.35	0.35	0.45	0.45
*Analyzed composition*				
Ash, %	5.63	5.59	6.82	6.90
Crude protein, %	14.5	14.5	20.5	21.5
Crude fiber, %	2.22	2.33	2.71	3.03
Crude fat, %	3.16	5.39	5.48	7.73
Moisture, %	4.55	4.44	4.54	3.85
Lysine, %	0.65	0.72	1.17	1.30
Methionine, %	0.22	0.23	0.31	0.32
Cysteine, %	0.22	0.24	0.32	0.33
Threonine, %	0.50	0.53	0.78	0.84
Tryptophan, %	0.20	0.19	0.30	0.32
Isoleucine, %	0.59	0.63	0.94	1.02
Leucine, %	1.37	1.40	1.82	1.89
Valine, %	0.67	0.71	1.02	1.10

^1^Diets were fed approximately 100 d of gestation until weaning.

Abbreviations: ATTD, apparent total tract digestible; SID, standardized ideal digestible.

All sows were exposed to HS conditions beginning 4 ± 1 d before expected farrowing. To apply HS environment, the room temperature was gradually increased from 29 to 32 °C with hourly increments of 1 °C from 0800 to 1100 hours and kept at 32 °C for 5 h then gradually reduced every hour from 1700 to 2000 hours until 26 °C. The farrowing house ventilation system was programmed to automatically increase speed and airflow with rise in room temperatures. The set point for the fan controller was also adjusted so that the fans operated at a minimum flow rate of 20% of their maximum capacity. This setting was implemented to maintain the target temperature while using heating systems without exceeding it. Farrowing room temperature, dew point, and relative humidity were recorded with data loggers (Lascar Electronics ELUSB2 EL-USB-2 Humidity, Temperature and Dew Point USB Data Logger; Erie, PA, USA) every 10 min during the entire experimental periods of replicate 1 and replicate 2 (Supplementary Figure 1).

Sow physiological responses to the HS environment were monitored by measuring respiration rates and rectal temperatures throughout the day and findings of this larger study are described in Stansberry et al. (2024). To cool the ECP groups, Hog Mat Coolers (Innovative Heating Technologies; Oak Bluff, MB, Canada) were installed on the floor of the farrowing crates. Conductive cooling was achieved by flushing water out of the pad when the base of the pad reached 26 °C (Cabezón et al., 2017). A sow with low colostrum production was removed a day after farrowing and another with severe dystocia was removed at farrowing. All sows received antibiotics treatment with no improvement before removal from the study. A sow refused to eat the Moringa diet at the time of assignment and was switched to a control diet and performed normally. The withdrawal of animals and reassignment of diet resulted in the final distribution of sows in each treatment-diet group across both replicates as follows: HS + CS (*n* = 12); HS + M (*n* = 11); ECP + CS (*n* = 13); and ECP + M (*n* = 10).

### Milk Collection

Colostrum was manually collected from all available teats during active farrowing of the sow when oxytocin is naturally high within 2 h of the birth of the first piglet into a 50 mL conical tube. Mature milk samples were manually collected on day L14 between 0600 and 1300 hours. To stimulate milk letdown, 0.5 cc of oxytocin (Bimedia with 20 usp/cc) was administered into the ear vein using a butterfly needle (Scalp vein set/butterfly needle 21G 3/4’) or 1 cc oxytocin into the vulva with a tuberculin syringe. Samples were apportioned into multiple aliquots, and temporarily stored at −20 °C and then transferred to −80 °C until analysis.

### Blood Collection

Using a 21G butterfly scalp vein set, 3 mL of blood was collected from the ear vein of sows into serum separation tubes on G111 or 112, L14 and L20. Blood was allowed to clot in the tube at room temperature for approximately 30 min, and then centrifuged at 500 × *g* for 15 min. Serum was divided into aliquots by pipetting 0.1 mL into 1 mL microtubes, and stored temporarily at −20 °C, then moved to −80 °C until analysis.

At PN1 and PN20, two median sized male and female piglets were randomly selected from each litter and approximately 1 mL of blood was collected from the jugular vein of the piglets into serum blood collection tubes using 21G syringe or vacutainer needles. Serum was separated and pooled by sex, and temporarily stored at −20 °C, and then transferred to −80 °C until analyzed.

### Fecal Collection

Representative fecal samples were obtained from 2 male and 2 female piglets per sow at PN14 using Rectal swabs (Puritan 25806 10WC, USA) during the second trial in October. Fecal swabs were placed in 5 mL sterile tubes and flash frozen in liquid nitrogen, then transferred to −80 °C.

### Cytokine Level Analysis

Milk cytokines were analyzed using a Porcine Cytokine Array C1 (RayBiotech, GA, US; catalog number AAP-CYT-1-8). Whey was isolated by centrifuging thawed whole milk samples at 12,000 × *g* for 30 min at 4 °C. The whey sample was collected from the bottom layer with a pipette after scooping the top fat layer with a spatula and pooled by treatment and study replicate to create a 1 mL working sample for each treatment group within the August or October replicate of the study. Samples were temporarily stored at −20 °C for <24 h before cytokine analysis. Within each study replicate and day of lactation, 4 sows were selected for the cytokine analysis, to correct for missing samples, and maintain an equal number of samples per treatment.

Analysis of levels of cytokines was completed following manufacturer’s protocol for the Porcine Cytokine Array C1 Kit (RayBiotech, GA, US). Briefly, the 16 membranes (2 study replicates × 4 treatment pools × 2 lactation days) were placed in wells and incubated with pooled samples, and then pools were incubated with biotinylated antibody cocktail, followed by another incubation step with HRP-Streptavidin. The membranes were then subjected to chemiluminescence detection using Bio-Rad Image Lab Software (Rio-Rad Laboratories 2017, 6.0.1 Build 34 Standard Edition) to quantify spot density.

To determine fold change, spot densities of cytokines on membranes were calculated using the outlined method in the Porcine Cytokine Array Kit protocol. Data were presented as the fold-difference between treatment from the control group: HS + CS within each lactation day. The differences were averaged between August and October replicates of the study, and the obtained values (Supplemental Table) were then transformed to Log_2_ using Microsoft Excel for Microsoft 365 (Version 2304 Build 16.0.16327.20200). Data presented in the figures were grouped by functions of cytokines-factors such as angiogenesis, cell adhesion molecule, chemokines, differentiation and growth, growth factor, hematopoiesis, hormone, interferons, interleukins, tumor necrosis factors, and other cytokines.

### Serum Analysis for Oxidation Status

Derivatives of Reactive Oxygen Metabolites (dROMs) and Biological Antioxidant Potential (BAP) were assessed in serum samples from sows (at G112, 14 L, and approximately 21 L) and piglets (at PN1 and PN20) using the Free Radical Elective Evaluator (FREE) Duo System by Diacron International (Grosseto, Italy) per the manufacturer’s instructions. An Oxidative Status Index (OSi) was calculated by dividing dROMs by BAP and then multiplying by 100 and the unit was recorded as U-CARR/µM. This index was used to quantify oxidative stress for both sows and piglets. dROMs functioned as an indicator of oxidative stress by measuring the conversion of organic hydroperoxide into radicals that oxidize N, N-diethyl-p-phenylenediamine, leading to a color change quantified using a spectrophotometer in U-CARR units. Conversely, BAP quantified the reduction of ferric ions to ferrous ions in the serum. The color-developing concentration was measured using a spectrophotometer, and the results were recorded in µM.

### Fecal Calprotectin Analysis

Neonate fecal samples were analyzed for levels of calprotectin (Supplemental Table), a biological measure of intestinal inflammation. All individual swab samples present in a 5 mL tube were thawed at room temperature. Afterward, 100 µL of 1× PBS was added to each swab sample and briefly vortexed, and then centrifuged at 1,000 × *g* for 20 min at 25 °C to pellet solids. To eliminate potential sex effects, equal volumes of male (*n* = 2/litter) and female (*n* = 2/litter) fecal extracts were pooled within a litter to create a working sample solution of approximately 100 µL. Afterward, samples were assayed as instructed in Porcine Calprotectin ELISA Kit (MyBioSource, San Diego, CA, USA; Catalogue number; MBS033848). All samples and standards were measured in duplicate.

### Statistical Analysis

SAS On Demand for Academics (SAS Institute Inc, Release 3.1.0, NC, USA) was used to analyze dependent variables of BAPs, dROMs, and OSi in sow milk and serum, and piglet serum (Supplemental Table). The PROC MIXED model evaluated fixed effects of cooling, diet, replicate, day, parity, and interaction effect of cooling by diet with sow as a random effect. The effect of sex and day (PN1 and PN20) was included in the piglet serum variable analysis. Least squares means and posthoc Tukey’s test were obtained, and statistical significance was determined at *P* < 0.05.

## Results

Our previous analysis of the effects of ECP and Moringa treatments on sow performance and litter growth found that ECP significantly (*P* < 0.05) decreased sow respiration rate and rectal temperature, and lactation body weight loss while increasing lactation feed intake (Stansberry et al., 2024). Moringa supplementation increased percent fat in milk. However, neither Moringa nor ECP affected number of piglets born, number born alive, number weaned, or litter weaning weight.

### Impact of Cooling and Moringa on Oxidative Status of Sows and Piglets

**Figure 1.**
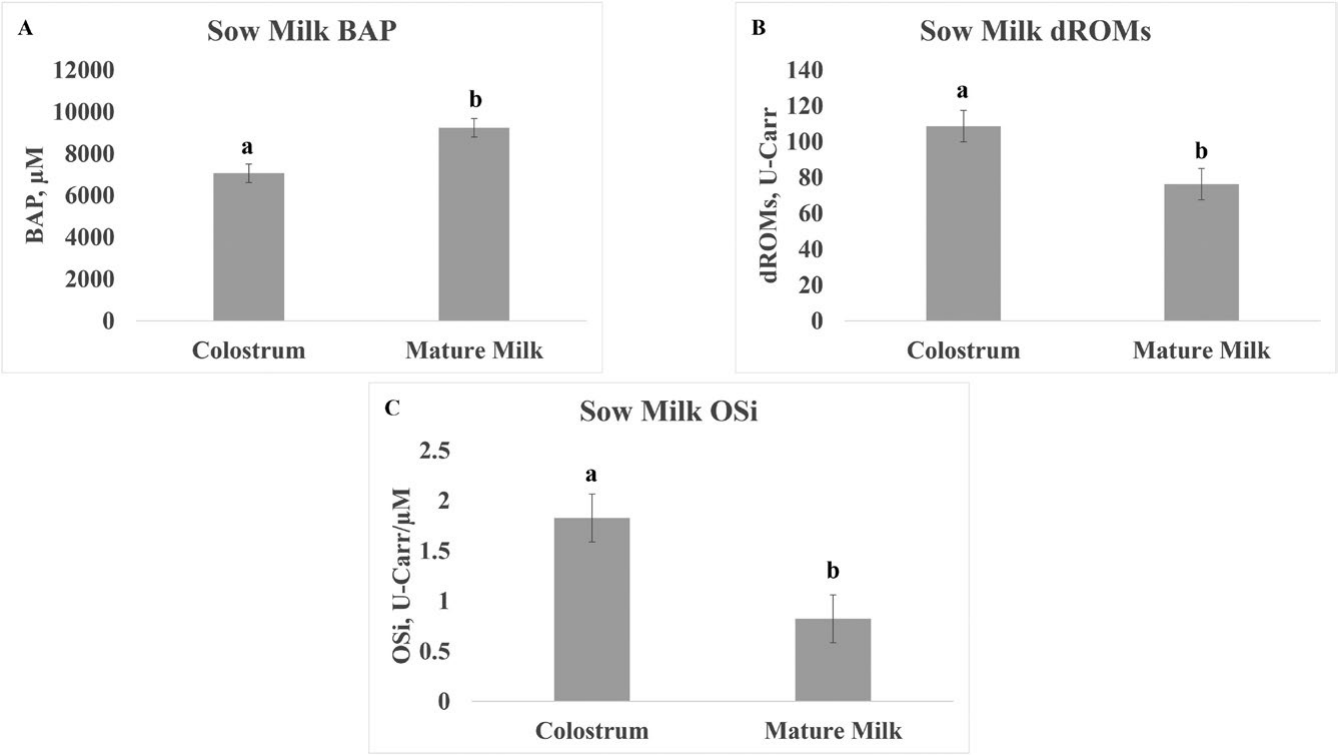
The effect of day of lactation on BAP (A), dROMs (B), and OSi (C) in colostrum and mature milk of sows. Whey fraction of milk from all sows was used for analysis and values are least squares means from PROC MIXED analysis in SAS with standard error of mean. Different letters indicate significant difference in variable between day of lactation at *P* < 0.05. Abbreviations: BAP, biological antioxidant potential; dROMs, derivative reactive oxygen metabolites; ECP + CS, cooled + corn–soybean diet; ECP + M, cooled + Moringa diet; HS + CS, heat stressed + corn–soybean diet; HS + M, heat stressed + Moringa; OSi, oxidative stress index (dROMs/BAP * 100).

Neither cooling nor diet affected sow serum BAP levels. However, there was a significant interaction (*P* = 0.029) between diet and cooling on BAP levels of sows, with Moringa increasing BAP levels in HS sows and decreasing BAP levels in cooled sows (Table 2). Diet significantly affected dROMs in sow serum (Table 2; *P* = 0.004), levels of dROMs were significantly higher levels in Moringa-supplemented animals than sows on control diets. The oxidative stress index (OSi) of sow serum levels was also significantly affected by diet (*P* = 0.04). The primary effect was within the ECP group, as indicated by the cooling by diet interactions, and the significantly lower OSi levels in serum of the ECP + CS group relative to EPC + M, HS + CS, and HS + M. Cooling also showed a tendency to reduce OSi in sow serum (*P* = 0.062). Stage of lactation significantly affected levels of BAP (*P* < 0.0001), dROMs (*P* = 0.001), and OSi (*P* = 0.0002) in sow milk (Table 2) with higher concentrations of BAP in mature milk than colostrum but higher concentrations of dROMs and OSi in colostrum than in mature milk (Fig. 1). There was no effect of treatment in any measured parameter of milk oxidation status.

**Table 2:** Oxidation status of sow serum and milk, and piglet serum across the August and October trial

		LSMEANS				SEM	*P*-values					
		ECP + CS	ECP + M	HS + CS	HS + M		Cooling	Diet	Rep	Day	Sex	Cooling*diet
Sow serum	BAP (U-Carr)	2,961.72	2,766.43	2,562.02	3,003.84	141.77	0.569	0.387	<0.0001	0.231	—	0.029
	dROMs µM	397.99	560.83	487.87	565.23	39.08	0.232	0.004	0.001	0.317	—	0.279
	OSi (U-Carr/µM)	13.39	20.10	19.80	19.15	1.44	0.062	0.040	0.979	0.226	—	0.013
Sow milk	BAP (U-Carr)	7,757.92	8,026.22	8,422.03	8,399.63	458.89	0.268	0.789	0.016	<0.0001	—	0.754
	dROMs µM	93.46	100.33	96.23	80.00	10.42	0.408	0.654	0.019	0.001	—	0.275
	OSi (U-Carr/µM)	1.35	1.77	1.17	1.02	0.25	0.075	0.590	0.248	0.0002	—	0.268
Piglet serum	BAP (U-Carr)	2,191.44	2,032.47	2,090.67	2,223.98	71.54	0.525	0.859	<0.0001	<0.0001	0.008	0.042
	dROMs µM	276.72	288.11	288.83	291.91	20.67	0.703	0.727	0.444	<0.0001	0.107	0.840
	OSi (U-Carr/µM)	12.06	13.62	13.69	12.44	0.96	0.818	0.875	0.0004	<0.0001	0.003	0.145

Abbreviations: BAP, biological antioxidant potential; dROMs, derivative reactive oxygen metabolites; ECP + CS, cooled + corn–soybean diet; ECP + M, cooled + 4% Moringa diet; HS + CS, heat stressed + corn–soybean diet; HS + M, heat stressed + 4% Moringa diet; LSMeans, least squares means; OSi, oxidative stress index; SEM, standard error of mean.

**Figure 2.**
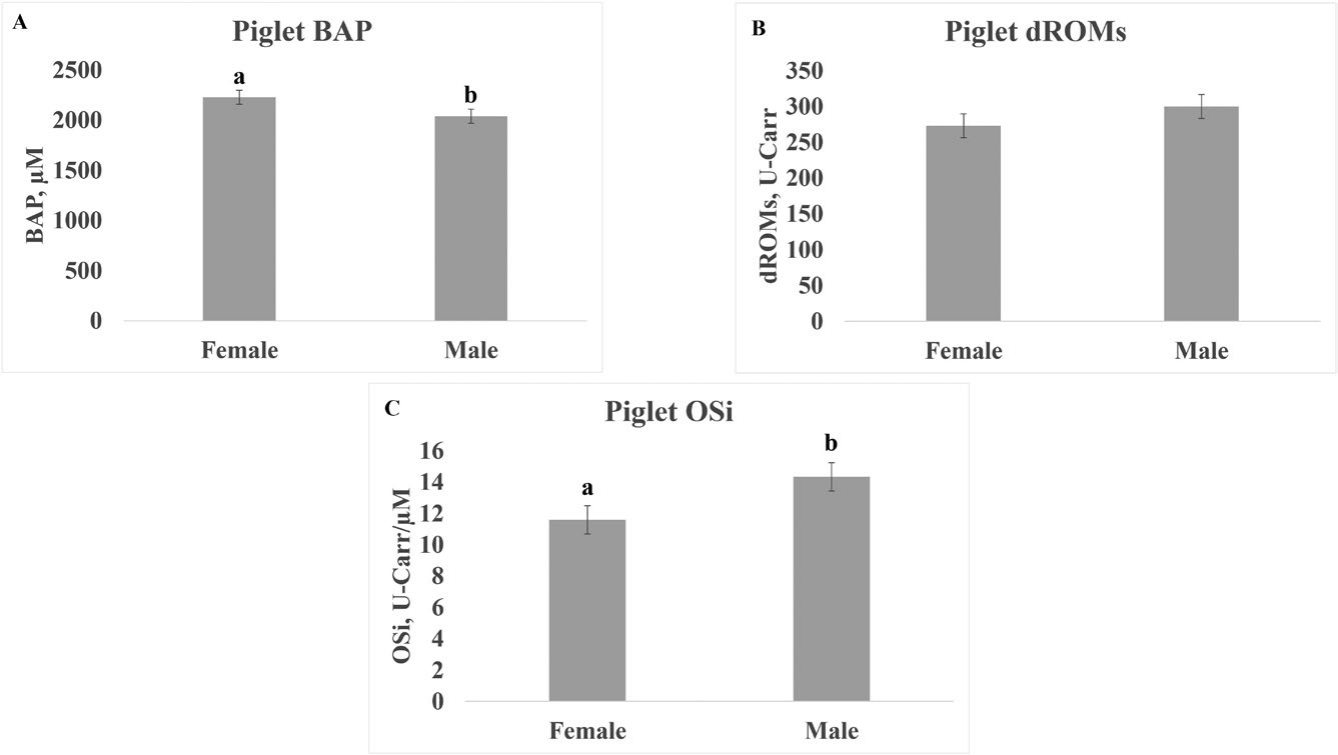
The effect of sex on levels of BAP (A), dROMs (B), and OSi (C) in serum of piglets. Samples were collected from 2 males and 2 females in each litter on postnatal days 1 and 20 and pooled by sex for each day. Values are least squares means from PROC MIXED analysis in SAS with standard error of mean. Different letters indicate significant difference in variable between sex at *P* < 0.05. Abbreviations: BAP, biological antioxidant potential; dROMs, derivative reactive oxygen metabolites; ECP + CS, cooled + corn–soybean diet; ECP + M, cooled + Moringa diet; HS + CS, heat stressed + corn–soybean diet; HS + M, heat stressed + Moringa; OSi, oxidative stress index (dROMs/BAP * 100).

**Figure 3.**
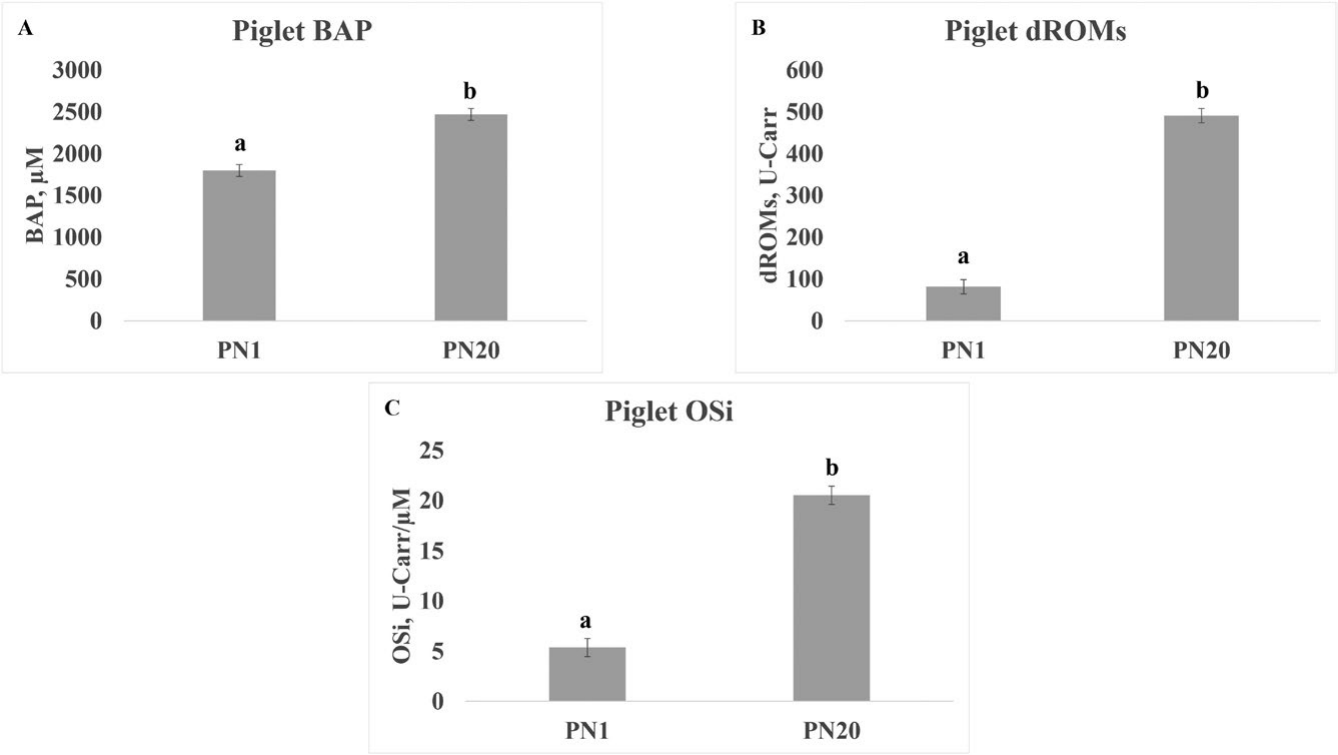
The effect of day on levels of BAP (A), dROMs (B), and OSi (C) in serum of piglets. Samples were collected from 2 males and 2 females in each litter on PN1 and PN20 and pooled by sex. Values are least squares means from PROC MIXED analysis in SAS with standard error of mean. Different letters indicate significant difference in variable between day postnatal at *P* < 0.05. Abbreviations: BAP, biological antioxidant potential; dROMs, derivative reactive oxygen metabolites; ECP + CS, cooled + corn–soybean diet; ECP + M, cooled + Moringa diet; HS + CS, heat stressed + corn–soybean diet; HS + M, heat stressed + Moringa; OSi, oxidative stress index (dROMs/BAP * 100); PN, postnatal.

Treatment did not affect piglet serum OSi levels (Table 2), but there was a significant interaction (*P* = 0.042) between cooling and diet on piglet serum BAP levels (Table 2). The highest concentration of BAP was in the serum of HS + M and ECP + CS piglets. Sex of piglets also significantly influenced BAP and OSi levels (*P* = 0.008 and 0.003) but no effect was found in levels of dROMS (Table 2). Levels of BAP were higher in females than males, and OSi was lower in females than in males (Fig. 2). Piglet levels of BAP, dROMs and OSi also varied significantly (*P* < 0.0001) by postnatal day (Table 2) with higher OSi at postnatal day 20 than day 1 across all measured parameters of BAP, dROMs and OSi (Fig. 3).

### Impact of Cooling and Moringa on Milk Cytokines

**Figure 4.**
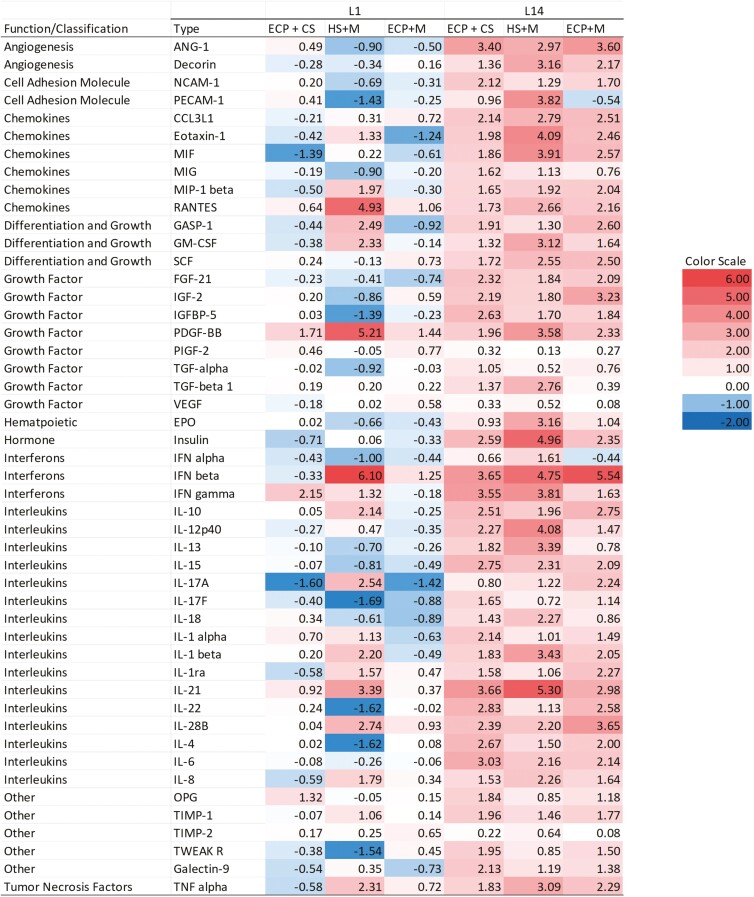
The impact of cooling and Moringa on the levels of cytokines in colostrum (L1) and mature milk (L14) in ECP + CS, ECP + M and HS + M relative to HS + CS. Milk samples were pooled by treatment on each day and used to probe cytokine arrays. Values presented are Log_2_ conversion of cytokine fold change calculated from the spot density of each treatment group divided by the spot density of the control group (HS + CS). The scale indicates relative changes in cytokine levels compared to the control within the L1 or L14. Values less than −1.0 correspond to a fold change of less than 0.5, while values greater than 1.0 correspond to a fold change of greater than 2 for cytokines in the treated groups compared to the control group. Abbreviations: Ang, angiopoietins; CCL3L, chemokine (C-C motif) ligand 3-like; ECP + CS, cooled + corn–soybean diet; ECP + M, cooled + Moringa diet; eotaxin-CCL11, chemokine (C-C motif) ligand; EPO, erythropoietin; FGF, fibroblast growth factor; GASP, growth and differentiation factor-associated serum protein; GM-CSF, granulocyte-macrophage colony-stimulating factor; HS + CS, heat stressed + corn–soybean diet; HS + M, heat stressed + Moringa; IGF, insulin-like growth factor; IGFBP, insulin like growth factor binding protein; IL, interleukin; MIF, macrophage migration inhibitory factor; MIG, monokine induced by interferon-gamma/CXCL9; MIP, macrophage inflammatory protein-CCL4; NCAM, neural cell adhesion molecule; OPG, osteoprotegerin; PDGF, platelet derived growth factor subunit B; PECAM, platelet and endothelial cell adhesion molecule; PIGF, placental growth factor; ra, receptor; RANTES, Regulated on Activation, Normal T Cell Expressed and Secreted; SCF, stem cell factor; TIMP, tissue inhibitor of metalloproteinases; TWEAK R, tumor necrosis factor-like weak inducer of apoptosis receptor; VEGF, vascular endothelial growth factor.

In colostrum (L1), levels of MIF were −1.39-fold and IL-17A −1.60-fold lower in ECP + CS than HS + CS. Colostrum of ECP-treated sows had 1.71, 2.15-fold greater levels of PDGF-BB and IFN gamma, respectively, than HS sows (Fig. 4). Relative to HS + CS, HS + M treatment had −1.0, −1.69, −1.62, −1.62-fold lower levels of INF alpha, IL-17F, IL-22, and IL-4. Moringa fed sows also had 1.33, 1.97, 4.93, 5.21, 6.10, 1.32, 2.14, 2.54, 1.13, 2.20, 1.57, 3.39, 2.74, 1.79, and 2.31 greater fold levels in Eotaxin-1, MIP-1 beta, RANTES, PDGF-BB, IFN beta, IFN gamma, IL-10, IL-17A, IL-1 alpha, IL-1 beta, IL-1ra, IL-21, IL-28B, IL-8, and TNF alpha than the HS group (Fig. 4). In colostrum (L1), levels of Eotaxin-1 were −1.24-fold and IL-17A −1.42-fold lower in ECP + M than HS + CS. RANTES, PDGF-BB, and IFN beta also had 1.06, 1.44, and 1.25-fold greater levels in colostrum of ECP + M than HS sows (Fig. 4).

In mature milk (L14) the treated groups ECP + CS, HS + M, and ECP + M had higher fold changes in all groups of cytokines examined (Fig. 1). ECP + CS sows had 3.66, 3.55, 3.66, and 3.03-fold higher levels in IFN beta, IFN gamma, IL-21, and IL-6 respectively of mature milk than the HS group (Fig. 1). Moringa fed sows also had greater 4.09, 3.91, 4.75, 3.85, 4.08, 3.39, 5.30-fold levels in Eotaxin-1, MIF, IFN beta, IFN gamma, IL12p40, IL-13, IL-21 than HS of mature milk. Levels of IFN beta were also 5.54-fold higher in mature milk of ECP + M sows than HS (Fig. 1).

### Effect of Cooling and Moringa on Intestinal Inflammation of Piglets

**Figure 5.**
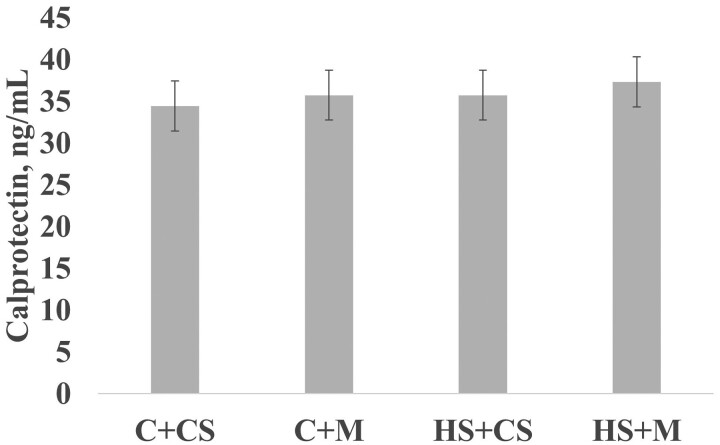
The concentration of calprotectin in piglets on PN14. Feces were collected from 2 males and 2 females in each litter on PN14. To eliminate potential sex effects, equal volumes of male and female fecal extract were pooled within a litter. Calprotectin was measured in fecal extracts by ELISA. Values are least squares means from PROC MIXED analysis in SAS with standard error of mean. Abbreviations: ECP + CS, cooled + corn–soybean diet; ECP + M, cooled + Moringa diet; HS + CS, heat stressed + corn–soybean diet; HS + M, heat stressed + Moringa.

Calprotectin levels of feces obtained from male and female piglets in the October trial were not significantly affected by treatment at PN14 (Fig. 5).

## Discussion

HS poses a significant economic challenge to the US swine industry, particularly regarding production losses of gestating and lactating sows and their litters. In late gestation and throughout lactation, sows are particularly vulnerable to HS due to the increased metabolism needed to support these physiological states (Lucy and Safranski, 2017; Mayorga et al., 2019). This study’s overall aim was to determine whether cooling sows in an HS environment with and without Moringa leaf dietary supplementation would improve inflammation and oxidation status of the sows and their piglets. Our overall findings indicate that both cooling and Moringa improved inflammatory cytokine levels in sow colostrum but not mature milk. While cooling improved oxidation status of sow serum, no treatment impacted piglet oxidative index or intestinal inflammation. Contrary to our hypothesis, Moringa potentially increased oxidative stress as indicated by higher OSi in sow serum.

Cooling or Moringa treatments increased the levels of antioxidants in the sow serum as sows on cooling treatment or Moringa had the highest BAP levels in their serum. We however noted an unexpected contradictory effect of Moringa to potentially increase oxidants with high dROMS observed in serum of HS + M treated sows. The increased BAP levels by Moringa corroborate general literature on Moringa’s antioxidant abilities and its capacity to scavenge free radicals (Alba et al., 2021; Yao et al., 2021). However, simultaneous elevation of reactive metabolites reflected by high dROMS is unexpected and contradicts previous research results. It also differs from the findings of (Sun et al., 2020), which reported that 4% Moringa in sow peripartum diets increased total antioxidant capacity and reduced malondialdehyde levels—a marker of oxidation. However, it is worth noting that the sows in their study were not heat-stressed. The anomaly in Moringa’s antioxidant role in this study remains unexplained. Lack of analysis of the phytochemical properties of the Moringa leaf precludes too much speculation, but it is known that flavonoids and polyphenols, which are commonly reported to be components identified in Moringa leaf (Chiș et al., 2023), activate aryl hydrocarbon receptor signaling which activates both anti-inflammatory and antioxidant pathways (Sondermann et al., 2023). Since cooling best-reduced dROMS compared to Moringa, we observed low oxidative stress in ECP + CS compared to other groups, meanwhile, ECP + M had high oxidative stress levels possibly because of Moringa increasing oxidant concentrations in the serum. The reactive metabolite reduction potential by cooling found in this study corroborated the antioxidant effect of cooling strategy against HS noted by (Safa et al., 2019) with cooled HS cows postcalving had lower concentrations of malondialdehyde and free oxygen radicals in plasma compared to HS counterpart.

Although the FREE duo system is validated for measuring BAP, dROMS, and OSi in serum, we also used this to analyze these variables in milk. Data supports that oxidative index in milk changes during lactation with higher levels of OSi in colostrum than in mature milk. HS also elevated the OSi levels in colostrum. This finding is similar to (Blond et al., 2024) reports of early lactation cows having a greater response to HS with higher levels of heat shock protein 70 than mid-lactation cows (Blond et al., 2024). Thus, the higher OSi in colostrum found in our study could be reflective of the high response of early lactation sows to HS and increased metabolism and stress associated with periparturient period (He et al., 2019; Lauridsen, 2019). Similarly, reactive oxygen species were higher in sows in the periparturient period than later in lactation, and supplementing pigs with oregano oil, as a source of antioxidants, during gestation to lactation found the treatment’s effect to mitigate oxidation was more pronounced at day 1 of lactation than at other timepoints through gestation and lactation (Tan et al., 2015). This finding also provides further insight regarding observations of treatment responses in our study, which also exhibited more impact on modulating inflammatory cytokines in the colostrum than in mature milk. These findings suggest late gestation period in sows as a critical focus for therapeutic strategies aimed at combating HS. Targeting this specific time frame could help prevent the detrimental effects on colostrum quality.

Several findings emerged in this study regarding the effects of treatments and stage of lactation on cytokine levels in milk. We observed that cooling and Moringa had varied independent and combined effects on cytokine levels in colostrum. Cooling reduced MIF levels in colostrum. MIF is a chemoattractant known to draw cytokines to the site of inflammation. Cooling also reduced colostrum IL-17A levels, which have pro-inflammatory functions that promote inflammation (Szulimowska et al., 2023). Our study also identified the combined effect of cooling and Moringa on colostrum to modulate inflammation by downregulation and upregulation of chemokines and other inflammation mediators as observed with decreased Eotaxin-1 and IL-17A, and increased RANTES, PDGF-BB, and IFN beta in ECP + M than HS group. While our study relays modulatory effects of cooling, Moringa, and cooling with Moringa, it is difficult to conclude on the treatment group with optimal modulatory effect, but worthy of note is the Moringa group among other treatments, with an observed upregulation of anti-inflammatory cytokines, IL-1ra, and IL-10. Alongside known phytochemical and medicinal properties of Moringa, our findings further support its potential in inflammation modulation and prompt further research.

Conversely in mature milk, we noted a consistent time-dependent pattern in the effects of treatments on cytokine levels in mature milk. Relative to HS + CS, inflammatory cytokines were greater in the other treatment groups. A study of HS response of cows in early and mid-lactation (Blond et al., 2024) in May, June, and July representing thermoneutral, mild, and severe HS periods, found early lactation cows to have higher response to HS than mid-lactation cows which corroborates the potential adaptation found in the HS group reflected in lower mature milk cytokines (Blond et al., 2024). As such, our findings indicate that mitigative strategies against HS effects should be directed to late gestation and early lactation when the sows are more prone to HS as the cytokine population is variedly affected by HS during this period. It may also indicate that an acute approach against HS conditions is more impactful than a long-term approach. Using cytokine arrays on pooled samples allowed us to test multiple cytokines at one time, however, the pooling of samples resulted in the loss of statistical power. These data will need verification in individual samples using other assays, like Enzyme-Linked Immunosorbent Assay, but here they provide foundational information for future exploration in related studies.

The impact of maternal HS during late gestation and lactation has both short and long-term effects on piglet growth and development (Lucy and Safranski, 2017; Liu et al., 2022). The detrimental effects of HS are attributed to maternal environment affecting offspring nutritional and developmental environment. Lower sow milk production often caused by HS is believed to be a primary means of negatively impacting litter growth. It is also highly probable that maternal oxidative stress and inflammation affect litter growth and piglet development. Here we collected blood from 2 males and 2 females from each litter to serve as sentinels for the exploration of the impact of treatments on markers of oxidative state. Our results indicate that cooling sows or feeding Moringa can influence concentrations of antioxidant potential in the blood circulation of the piglets. Piglets in litters of ECP + CS and HS + M sows, had higher levels of BAP in serum, indicating that antioxidant levels in piglets mirror that of their dams. Our finding is supported by others that reported antioxidant markers of total antioxidant status and levels of superoxide dismutase in piglet serum mirrored those of the sow at day 21 of lactation (Lipiński et al., 2019). However, circulating dROMs and OSi in piglet serum did not reflect levels circulating in sow serum. Rather piglet oxidation status was more profoundly affected by age, with OSi increasing from PN1 to PN20. This finding may indicate that over time the circulating oxidants and antioxidants in the piglet are primarily generated by the piglet itself from increased metabolism associated with growth and are less dependent on the sow status (Martemucci et al., 2022). Our previous analysis of these study animals found that neither ECP nor Moringa supplementation affected a number of piglets born, the number born alive, the number weaned, or the final litter weight (Stansberry et al., 2024). The lack of a difference in weaning litter size and weight indicates that treatments did not significantly impact milk production levels, and sow oxidative stress may not directly impair litter growth during lactation.

Also noteworthy, although litter was the experimental unit of the described studies, analysis of male and female piglets separately enabled the revelation that piglet oxidation status varied between males and females. These data highlight the importance of considering sex as a biological variable, with a recent study conducted by (Mayorga et al., 2024) demonstrating gilts respond differently to antioxidant dietary supplementation compared to barrows under HS conditions.

There was also an effect of the study replicated on oxidation status measures in sows and piglets. It is likely that multiple factors contributed to differences between August and October replicates such as the differences in exposure to previous HS conditions and photoperiod. The sows in the August trial would have experienced a higher ambient temperature prior to the study than that of the sows in the October trial. We acknowledge that prior HS exposures could modify reaction of sows and adaptation to the HS environmental condition as either chronic or acute response and variably impact results. To help mitigate this limitation, sows in both trials were moved into the experimental rooms two weeks pre-heat treatment during this 2-wk period the ambient temperatures were controlled and maintained similarly. However, although room temperature could be controlled for application of moderate HS conditions, relative humidity could not be controlled. HS tolerance is affected by temperature-humidity index, and both high temperature and high temperature-humidity index affect production performance of sows (Wegner et al., 2016).

In this study, we found no impact of any of the treatments on intestinal inflammation in the piglets at PN14 born to HS sows reflected in indifferent levels of calprotectin measures of intestinal inflammation. The lack of a treatment effect on intestinal inflammation is consistent with the lack of a treatment effect on serum oxidative status of piglets. Additionally, to be considered was the finding that differences between treatments in cytokine levels indicate that compared to HS + CS nearly all cytokines were greater in mature milk of HS + M, ECP + CS, and ECP + M sows. These differences in milk cytokines were thus unrelated to calprotectin levels in neonate gut.

Together these findings demonstrate that both cooling and Moringa modified inflammatory cytokine levels in sow colostrum, and cooling further improved the oxidation status in serum of sows while piglet intestinal inflammation and oxidative status were unaffected by HS exposure of sows. Oxidative factors also varied by stage, with greater oxidative stress indexes associated with early lactation than late lactation as observed in milk. Piglets also exhibited a greater oxidative status at 3 wk of age versus immediately after birth, indicating that developing litters are prone to oxidative stress during growth and increased metabolism. These distinct effects of cooling and Moringa, as well as stage differences in oxidative variables, should be considered when testing and designing research trials and potential therapeutic interventions, with the periparturient period in sows seemingly a period of high oxidative index and inflammation, and thus an ideal time to target against HS.

## Supplementary Material

txae156_suppl_Supplementary_Figure_S1

txae156_suppl_Supplementary_Tables
