# Supplementary figures and images for: The impact of cooling and Moringa supplementation on oxidative stress in serum and milk, including milk cytokines, in heat stressed lactating sows and their litters
Source: Transl Anim Sci. 2024 Nov 13;9:txae156. doi: 10.1093/tas/txae156 (PMC11751637; doi:10.1093/tas/txae156)

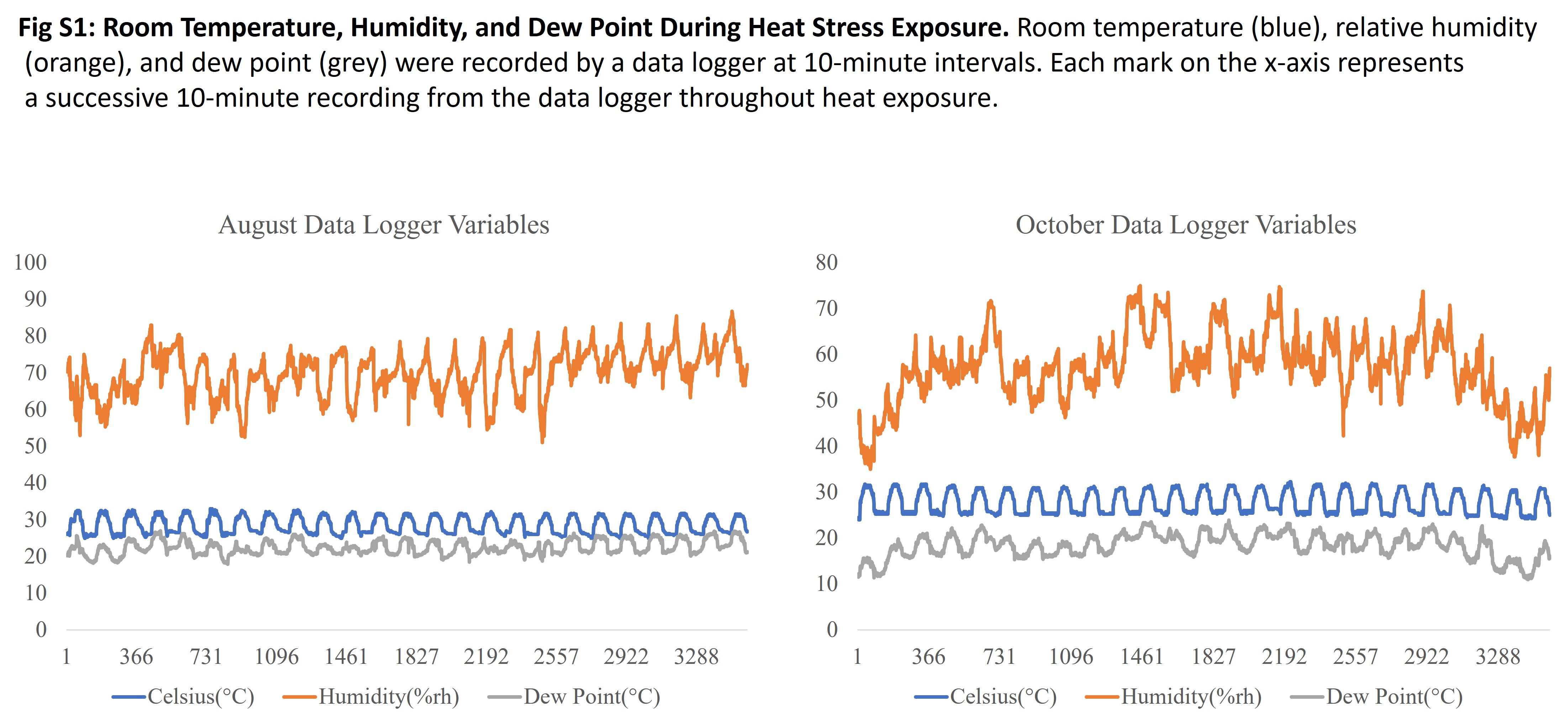

Supplement: txae156_suppl_Supplementary_Figure_S1 [file txae156_suppl_supplementary_figure_s1.jpeg]
